# Factors associated with lumbar spinal stenosis in a large-scale, population-based cohort: The Wakayama Spine Study

**DOI:** 10.1371/journal.pone.0200208

**Published:** 2018-07-18

**Authors:** Takahiro Maeda, Hiroshi Hashizume, Noriko Yoshimura, Hiroyuki Oka, Yuyu Ishimoto, Keiji Nagata, Masanari Takami, Shunji Tsutsui, Hiroshi Iwasaki, Akihito Minamide, Yukihiro Nakagawa, Yasutsugu Yukawa, Shigeyuki Muraki, Sakae Tanaka, Hiroshi Yamada, Munehito Yoshida

**Affiliations:** 1 Department of Orthopaedic Surgery, Wakayama Medical University, Wakayama City, Wakayama, Japan; 2 Department of Preventive Medicine for Locomotive Organ Disorders, 22nd Century Medical and Research Center, Faculty of Medicine, The University of Tokyo, Bunkyo-ku, Tokyo, Japan; 3 Department of Medical Research and Management for Musculoskeletal Pain, 22nd Century Medical and Research Center, Faculty of Medicine, The University of Tokyo, Bunkyo-ku, Tokyo, Japan; 4 Department of Orthopaedic Surgery, Faculty of Medicine, The University of Tokyo, Bunkyo-ku, Tokyo, Japan; 5 Department of Orthopaedic Surgery, Sumiya Orthopaedic Hospital, Wakayama City, Wakayama, Japan; Medical College of Wisconsin, UNITED STATES

## Abstract

**Objective:**

Patients with lumbar spinal stenosis (LSS) who have radiographically similar degrees of stenosis may not necessarily exhibit equivalent symptoms. As part of a cross-sectional study, we examined factors associated with symptomatic LSS (sLSS) in the general population of Japan.

**Methods:**

We evaluated 968 participants (men, 319; women, 649) between 2008 and 2010. Orthopedic surgery specialists diagnosed sLSS using interview results, medical examinations, and imaging findings. LSS was radiographically graded using a 4-level scale. Additionally, we examined basic anthropometry, smoking habits, alcohol consumption, ankle-brachial index values (ABI), and glycosylated hemoglobin (HbA1c) levels. We grouped patients with moderate and severe radiographic LSS, and compared the indicated factors on the basis of the presence/absence of sLSS. Data were evaluated using multiple logistic regression analyses.

**Results:**

Radiographically, 451 participants had moderate and 288 severe stenosis. Clinically, 92 participants were diagnosed with sLSS, including 36 with moderate and 52 with severe stenosis. In the moderate stenosis group, participants with sLSS had significantly higher rates of diabetes mellitus (DM) and lower ABIs than did non-LSS participants. Although sLSS participants tended to be older (p = 0.19), there were no significant differences in the sex distribution, body mass index values, or in the percentages of participants who were drinkers/smokers. In the severe stenosis group, there were no differences in any of the evaluated factors. Multiple logistic regression showed that DM (odds ratio [OR], 3.92; 95% confidence interval [CI], 1.52–9.34]) and low ABI (1 SD = 0.09; OR, 1.36; 95% CI, 1.04–1.81) were significantly associated with LSS in the moderate stenosis group.

**Conclusions:**

DM and low ABIs are significantly associated with sLSS in patients with moderate radiographic stenosis. Neither factor is associated with sLSS in patients with severe stenosis. Notably, the effects of intrinsic factors on symptomology may be masked when anatomic stenosis is severe.

## Introduction

Lumbar spinal stenosis (LSS) is a narrowing of the spinal canal that results in the compression of the spinal cord and nerves. This compression results in pathognomonic symptoms such as pain, lower limb numbness, and intermittent claudication while walking or standing for long periods of time. Patients with symptomatic LSS (sLSS) generally demonstrate walking intolerance, various physical disabilities, disability in daily activities, and/or low quality-of-life scores compared with healthy individuals [1,2]. LSS is common in elderly patients because it is mainly caused by age-related degeneration and hypertrophy of the intervertebral disks, ligamenta flava, and facet joints. LSS is the most common reason for spinal surgery in patients >65 years old [3, 4]. However, many healthy individuals also demonstrate similar age-related changes, including similar degrees of radiographic LSS (rLSS) to that in patients with sLSS, but remain asymptomatic [5]. Recent studies have shown that lifestyle-related diseases such as diabetes mellitus (DM), peripheral artery disease (PAD), and heart disease, are frequently associated with sLSS [6–8]. However, the prevalence of medical comorbidities also increases with age, and to our knowledge, most previous studies were case-control studies with selection bias. Thus, definite conclusions have not yet been drawn. In addition to rLSS, PAD and DM are considered risk factors for sLSS, but the risk factors for sLSS have not been studied extensively. Unfortunately, there is no clear evidence regarding the potential risk factors for sLSS adjusted for rLSS, PAD and DM. On the basis of this absence of suitable data, we conducted a large-scale cohort study of the general Japanese population. Using data from that study, we examined the associations between the potential risk factors in individuals with sLSS adjusted for rLSS, PAD and DM.

## Methods

### Ethical considerations

All participants provided written informed consent, and the study was approved by the ethical committees of Wakayama Medical University and The University of Tokyo.

### Participants

The Wakayama Spine Study is a population-based study of patients with degenerative spinal disease performed using data from the Research on Osteoarthritis/osteoporosis Against Disability (ROAD) study, a large-scale nationwide prospective study of patients with bone and joint diseases consisting of population-based cohorts established in several communities throughout Japan. A detailed profile of the ROAD study has been previously described [9–13]. Briefly, a baseline database was created that includes the clinical and genetic information of 3040 individuals (1061 men, 1979 women), ranging from 23 to 95 years (mean, 70.6). Participants were recruited from listings of residents in three communities, including Itabashi, an urban region of Tokyo; Hidakagawa, a mountainous region of Wakayama prefecture; and Taiji, a coastal region of Wakayama prefecture. Participants completed a 400-item questionnaire administered by interviewers on lifestyle, anthropometric measurements, and physical performance measures.

**Fig 1:**
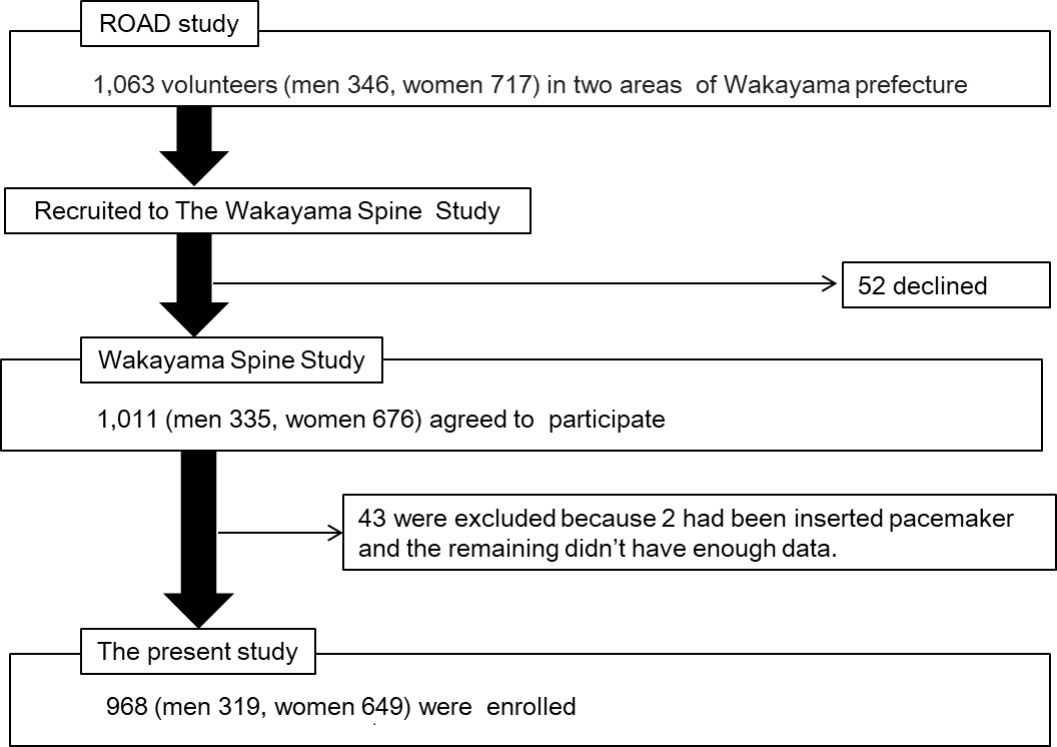
Flow diagram depicting participants recruited to the Wakayama Spine Study from the ROAD study.

A second visit to Hidakagawa and Taiji was performed between 2008 and 2010 during the ROAD study. Similar to that in the baseline study, participants completed a 400-item questionnaire administered by interviewers on lifestyle (e.g., smoking and alcohol consumption habits), family medical history, past medical history, physical activity, reproductive variables, and health-related quality of life information in this second visit. Anthropometric measurements included height, weight, bilateral grip strength, body mass index (BMI, weight [kg]/height [m]^2^), and ankle-brachial index (ABI; PWV/ABI, Omron, Kyoto, Japan) [14]. Comorbidities were defined using blood chemistry data (DM: glycosylated hemoglobin [HbA1c] ≥ 6.1% [15]. Among the individuals who participated in the second visit, 1063 volunteers were recruited to undergo MRI examinations and 52 declined. The remaining participants provided additional written informed consent for the MRI examinations. Among 1011 participants, MRIs were contraindicated in two individuals because of the presence of pacemakers, and 41 were excluded because of missing data. Thus, 968 participants (319 men, 649 women), ranging in age from 21 to 93 years were analyzed in the present study (Fig 1).

### Magnetic resonance imaging

A mobile magnetic resonance imaging (MRI) unit (Excelart 1.5 T, Toshiba, Tokyo, Japan) was used in our study. Each participant underwent a total spinal MRI on the same day as physical examination. The MRI exclusion criteria were the presence of a cardiac pacemaker, claustrophobia, or other contraindications. Participants were placed in a supine position during the MRI; those with rounded backs were supported using triangular pillows under their heads and knees. The protocol included sagittal T2-weighted fast spin echo (repetition time, 4000 ms/echo; echo time, 120 ms; field of view, 180 × 180 mm) imaging.

### Diagnosis of the symptomatic LSS

Experienced orthopedic surgeons reviewed patients’ medical history and performed physical testing for each study participant. Patient history included information on pain in the lower back, buttocks, and legs; the area of pain or other discomfort; the presence of intermittent claudication and its distance; and a modified Zurich Claudication Questionnaire [16], excluding the 6 items regarding satisfaction and history of lumbar surgery for sLSS. Physical examination included symptoms induced by lumbar extension, whether symptoms improved or were induced by lumbar flexion, floor–finger distance (cm), peripheral circulation (good or poor), straight-leg raising test, manual muscle testing of the upper and lower extremities, tendon reflex testing of the upper and lower extremities, and Babinski reflex testing.

The diagnostic criteria for sLSS were based on the North American Spine Society (NASS) guideline LSS definition, which requires the presence of LSS symptoms and radiographic signs of LSS [17]. Patients were diagnosed with LSS if they met the criteria described in Table 1. Symptom characteristics were induced or exacerbated by walking or prolonged standing and relieved by lumbar flexion, sitting, or recumbence.

**Table 1:** Diagnostic criteria for symptomatic LSS.

	Patients with symptomatic LSS met all of the criteria below:
1	Gluteal and/or lower extremity pain and/or numbness and/or fatigue, which may occur with or without back pain
2	Symptom provocation due to upright exercise, such as walking or standing (i.e., positional induced neurogenic claudication)
3	Palliative release of the symptoms with forward flexion, sitting, and/or recumbency
4	Radiological findings of spinal stenosis secondary to degenerative changes that can explain the symptoms

### Evaluation of the radiographic LSS

**Fig 2:**
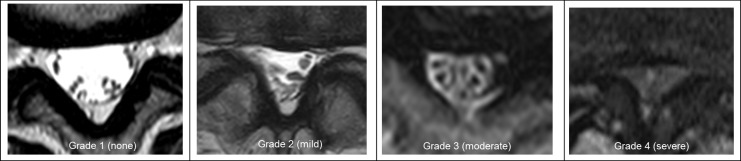
Lumbar spinal stenosis grading, based on magnetic resonance images. The general guideline classification was applied. Grade 1: mild stenosis as narrowing of the normal area by one-third or less, Grade 2: moderate stenosis as narrowing between one-third and two-thirds, Grade 3: severe stenosis as narrowing of more than two-thirds.

The severity of rLSS was assessed using qualitative measurements performed by a well-experienced orthopedic surgeon; axial images were provided on films. The severity of central stenosis was assessed according to the general guideline (Suri) classification [18], which is often cited by several reports, including this study (Fig 2). According to the Suri classification, rLSS was divided into 4 levels (Grade 0, no narrowing; Grade 1, mild: narrowing of < one-third of the normal area; Grade 2, moderate: narrowing of one-third to two-thirds of the normal area; Grade 3, severe: narrowing of > two-thirds of the normal area). To evaluate the intraobserver variability of the severity rating, 50 randomly selected lumbar MRI films were scored by the same observer more than 1 month after the first reading. Fifty other lumbar MRI films were also scored by two experienced orthopedic surgeons to determine the interobserver variability. The intraobserver variabilities in severity rating were confirmed by kappa analysis to be sufficient as 0.82; interobserver variability was also sufficient as 0.77.

### Statistics

We examined differences between men and women, the number of participants in each age group, ages, heights, weights, BMIs, HbA1c levels, ABIs (the lower of the ABIs determined with each leg), smoking habits, alcohol consumption, presence of DM, and presence of PAD using the Student's *t*-test. The Chi-square test was used to examine differences in sex, BMI, smoking habits, alcohol consumption, presence of DM, and the most severe rLSS grades between patients with and without LSS. Fisher's exact test was used to compare the presence/absence of PAD.

We performed multiple logistic regression analyses using the presence of sLSS as the objective variable, with explanatory variables including smoking habits, alcohol consumption, presence of DM, ABI, and most severe rLSS grade; moderator variables included sex, age, and BMI. We calculated the variance inflation factor using the least-squares method before running the regression analysis to confirm the absence of a strong correlation between variables.

We conducted a stratified analysis of individuals with radiographically moderate stenosis. Sex and alcohol consumption between those with and without LSS was evaluated using the Chi-square test; the prevalence of overweight, DM, and PAD, as well as smoking habits, using Fisher's exact test; and age, BMIs, HbA1c levels, and ABIs, was evaluated using Student's *t*-test. In addition, we used a multiple logistic regression analysis to determine the odds ratio for the association of age, DM, and ABI with sLSS. The same types of analyses were conducted for individuals with radiographically severe stenosis.

All statistical analyses were performed using JMP Pro 12.2 (SAS Institute Japan, Tokyo Japan). P-values < 0.05 were considered significant, and those from 0.05 to 0.10 were considered indicative of a significant trend.

## Results

Table 2 summarizes characteristics of the 968 participants (319 men and 649 women, mean age 66.3 years) including age and arthropometric participants. Prevalence of DM and PAD were 8.4% and 1.86%, respectively. The rates of smoking, alcohol consumption, DM, and PAD were higher among men than among women.

**Table 2:** Characteristics of the participants.

	Overall	Men	Women
	N = 968	n = 319	n = 649
**Age strata (y)**			
< 50, n	125	38	87
50–59, n	174	58	116
60–69, n	224	66	158
70–79, n	259	87	172
≥ 80, n	186	70	116
**Demographic characteristics (mean ± SD)**			
Age (y)	66.3 ± 13.5	67.0 ± 13.9	66.0 ± 13.3
Height (cm)	155.9 ± 9.4	164.7 ± 7.1	151.5 ± 7.2
Weight (kg)	56.8 ± 11.5	64.6 ± 11.6	53.0 ± 9.4
BMI (kg/m^2^)	23.3 ± 3.6	23.7 ± 3.4	23.1 ± 3.7
HbA_1_c (JDS %)	5.24 ± 0.71	5.28 ± 0.86	5.22 ± 0.62
ABI	1.10 ± 0.09	1.13 ± 0.10	1.09 ± 0.09
**Prevalence of selected characteristics (%)**			
Smoking	11.3	26	4.2
Alcohol consumption	32	58.3	19.1
Diabetes mellitus (HbA_1_c ≥ 6.1)	8.4	11.1	7.1
PAD (ABI < 0.9)	1.86	2.51	1.54

SD = standard deviation; BMI = body mass index; ABI = ankle-brachial pressure index; HbA1c = glycosylated hemoglobin; PAD = peripheral arterial disease; JDS = Japan Diabetes Society. PAD was defined as an ABI < 0.9 and diabetes mellitus as an HbA1c ≥ 6.1%. JDS values are approximately 0.4% lower than the NGSP values, which is the global standard.

Table 3 shows comparisons between sexes, ages, BMIs, HbA_1_c levels, DM (HbA_1_c **≥** 6.1), ABIs, PAD, smoking habits, and alcohol consumption between symptomatic and asymptomatic participants. sLSS was present in 92 (9.5%) participants, including 32 (10.0%) men and 60 (9.2%) women. Sex, age, BMI, smoking habits, alcohol consumption, and HbA_1_c levels were not significantly associated with sLSS. However, there were significant correlations with LSS for age, ABI, PAD, and the most severe rLSS grades on MRIs. The trend became stronger with increasing severity of the rLSS grade.

**Table 3:** Comparison between the characteristics in the symptomatic LSS and non-LSS group.

	Symptomatic LSS	Non-LSS	p-value
	n = 92	n = 876	
Sex (men:women)	32:60	287:589	0.695
Age (y)	71.6 ± 10.7	65.8 ± 12.5	**< 0.0001**
BMI (kg/m^2^)	23.5 ± 3.3	23.2 ± 3.6	0.5153
Overweight (BMI ≥ 25)	25 (27.2%)	256 (29.2%)	0.6803
Smoking habit	12 (13.0%)	97 (11.1%)	0.5776
Alcohol consumption	26 (28.3%)	284 (32.4%)	0.416
HbA1c (JDS %)	5.35 ± 0.79	5.23 ± 0.70	0.1066
Diabetes Mellitus (HbA1c ≥ 6.1)	16 (17.4%)	64 (7.3%)	**0.0009**
ABI	1.08 ± 0.10	1.10 ± 0.09	**0.0214**
PAD (ABI < 0.9)	6 (6.5%)	12 (1.4%)	**0.0045**
**Most severe rLSS grade between L1/2 and L5/S**			
Grades 0 and 1	4	225	**< 0.0001**
Grade 2	36	415	
Grade 3	52	236	

The chi-square test was used to clarify the differences between the LSS and non-LSS group in sex, overweight, smoking habit, alcohol consumption, diabetes mellitus, and rLSS grades. The Fisher's exact test was used to examine between-group differences in PAD and the Student's t-test was used for age, BMI, HbA1c, and ABI. LSS = lumbar spinal stenosis; BMI = body mass index; ABI = ankle brachial pressure index; PAD = peripheral arterial disease; rLSS = radiographic LSS. Overweight was defined as a BMI was ≥ 25, PAD as an ABI<0.9, and diabetes mellitus as an HbA1c (JDS) ≥ 6.1%. JDS values are approximately 0.4% lower than the NGSP values, which is the global standard. Significant p-values are indicated by bold type.

Multivariable logistic regression analysis (Table 4) showed that ABIs and the most severe rLSS grades were significantly associated with sLSS (ABI, p = 0.0431; most severe rLSS grade, p < 0.05). DM also tended to be associated with sLSS (p = 0.0585). The prevalence of LSS increased significantly with increasing LSS grade severity. There were no significant differences associated with sex, age, BMI ≥ 25, smoking habits, and alcohol consumption. The area under the curve for this model was 0.73.

**Table 4:** Association of selected factors with symptomatic lumbar spinal stenosis.

	Adjusted OR	95% CI	p-value
Sex (men, 1 vs women, 0)	1	0.58–1.71	0.9871
Age (+1 y)	3.54	0.82–16.3	0.2823
Overweight (yes, 1 vs no, 0)	0.79	0.47–1.31	0.3687
Smoking (yes, 1 vs no, 0)	1.89	0.87–3.90	0.107
Alcohol consumption (yes, 1 vs no, 0)	0.95	0.55–1.61	0.8517
Diabetes mellitus (yes, 1 vs no, 0)	1.89	0.98–3.50	*0*.*0585*
ABI (-1 SD)	1.24	1.06–1.52	**0.0431**
Most severe rLSS grade between L1/2 and L5/S			
Grade 2 vs Grades 0 and 1	4.31	1.66–14.7	**0.0016**
Grade 3 vs Grades 0 and 1	9.95	3.79–34.4	**< 0.0001**
Grade 3 vs Grade 2	2.31	1.44–3.73	**0.0005**

A multivariable logistic regression analysis was performed to determine the association between smoking, alcohol consumption, diabetes mellitus, ABI, and rLSS grade, and symptomatic LSS. OR = odds ratio; CI = confidence interval; ABI = ankle-brachial pressure index; rLSS = radiographic LSS. Overweight was defined as a BMI ≥ 25 and diabetes mellitus as an HbA1c (JDS) ≥ 6.1%. JDS values are approximately 0.4% lower than the NGSP values, which is the global standard. Significant p-values are indicated by bold type. The italics indicates trends toward significance.

We subsequently compared symptomatic and asymptomatic individuals with moderate rLSS. The prevalence of DM, ABI, and PAD was significantly different between the two groups (DM, p = 0.0009; ABI, p = 0.0026; PAD, p = 0.0281); there were no significant differences associated with the other factors (Table 5).

**Table 5:** Comparison of the characteristics exhibited by symptomatic and asymptomatic participants in those diagnosed with moderate radiographic lumbar spinal stenosis (LSS).

	Symptomatic LSS	Asymptomatic LSS	p-value
	n = 36	n = 415	
Age	69.6 ± 11.4	66.8 ± 12.5	0.1972
Sex (men:women)	12: 24	135: 280	0.9214
BMI	23.7 ± 3.9	23.1 ± 3.6	0.3569
Overweight (BMI ≥ 25)	9/36 (25.0%)	114/415 (27.5%)	0.7496
HbA_1_c (JDS %)	5.3 ± 1.0	5.2 ± 0.5	0.1983
Diabetes mellitus (HbA_1_c ≥ 6.1)	9/36 (25.0%)	26/407 (6.4%)	**0.0009**
ABI	1.06 ± 0.14	1.11 ± 0.09	**0.0026**
PAD (ABI <0.9)	3/36 (8.3%)	6/415 (1.45%)	**0.0281**
Alcohol consumption	10/36 (27.8%)	135/415 (32.5%)	0.5581
Smoking habit	6/36 (16.7%)	40/413 (9.7%)	0.2439

The Chi-square test was used to clarify the differences in sex and alcohol consumption between the LSS and non-LSS groups. The Fisher's exact test was used to examine between-group differences in BMI ≥ 25, diabetes mellitus, PAD, and smoking habits. The Student's t-test was used for age, BMI, HbA1c, and ABI. Data were not available for HbA1c (n = 8) and smoking habit (n = 2) in the asymptomatic LSS group. BMI = body mass index; JDS = Japan Diabetes Society; ABI = ankle brachial pressure index; PAD = peripheral arterial disease; HbA1c = glycosylated hemoglobin. Overweight was defined as a BMI ≥ 25, PAD as an ABI <0.9, and diabetes mellitus as an HbA1c ≥ 6.1%. Significant p-values are indicated by bold type.

DM and low ABIs were significantly associated with sLSS in this group on the basis of the results of a multiple logistic regression analysis evaluating the association between sLSS and age, DM (HbA1c ≥ 6.1), and PAD (Table 6).

**Table 6:** Association between age, diabetes mellitus, and ankle-brachial index values (ABIs), and symptomatic lumbar spinal stenosis in the participants with moderate radiographic stenosis.

	Odds ratio	95% CI	p-value
Age (+1 y)	1.02	0.99–1.05	0.3316
Diabetes mellitus	3.92	1.52–9.34	**0.0059**
ABI (-1SD)	1.36	1.04–1.81	**0.0271**

A multivariable logistic regression analysis was performed to determine the association between age, diabetes mellitus, and ABI, and symptomatic lumbar spinal stenosis in the in the participants with moderate radiographic stenosis. Diabetes mellitus was defined as HbA1c (JDS) ≥ 6.1%. JDS values are approximately 0.4% lower than the NGSP values, which is the global standard. Significant p-values are indicated by bold type.

There was no significant association between sLSS and age, sex, BMI, overweight, HbA1c, DM (HbA1c ≥ 6.1), ABI, PAD, alcohol consumption, or smoking habits in symptomatic and asymptomatic participants with radiographically severe LSS (Table 7).

**Table 7:** Comparison of the characteristics exhibited by symptomatic and asymptomatic participants in those diagnosed with severe radiographic lumbar spinal stenosis (LSS).

	Symptomatic LSS	Asymptomatic LSS	p-value
	n = 52	n = 236	
Age	72.8 ± 10.1	72.1 ± 11.0	0.6438
Sex (men:women)	19: 33	77: 159	0.5881
BMI	23.3 ± 3.1	23.7 ± 3.7	0.4077
Overweight (BMI ≧ 25)	14/52 (27.0%)	87/236 (36.9%)	0.1739
HbA1c (JDS %)	5.4 ± 0.6	5.5 ± 1.0	0.4198
Diabetes mellitus (HbA1c ≧ 6.1)	6/51 (11.8%)	30/233 (12.9%)	1
ABI	1.09 ± 0.10	1.11 ± 0.08	0.4049
PAD (ABI < 0.9)	3/52 (5.8%)	4/236 (1.7%)	0.1138
Alcohol consumption	14/52 (27.0%)	70/236 (30.0%)	0.6942
Smoking	6/52 (11.5%)	16/235 (6.8%)	0.2524

The chi-square test was used to clarify the differences in sex, overweight, and alcohol consumption between the symptomatic LSS and asymptomatic LSS groups. The Fisher's exact test was used to examine between-group differences in diabetes mellitus, PAD, and smoking habit. The Student's t-test was used for age, BMI, HbA1c, and ABI. Data were not available for HbA1c (n = 3) and smoking habit (n = 1) in the asymptomatic LSS group. BMI = body mass index; JDS = Japan Diabetes Society; HbA1c = glycosylated hemoglobin; ABI = ankle brachial pressure index; PAD = peripheral arterial disease. Overweight was defined as a BMI ≥ 25, PAD as an ABI < 0.9, and diabetes mellitus as an HbA1c (JDS) ≥ 6.1%. JDS values are approximately 0.4% lower than the NGSP values, which is the global standard.

## Discussion

### Key results

The purpose of this study was to uncover factors related to sLSS other than anatomic spinal canal stenosis. We found that the grade of MRI stenosis and low ABIs were significantly associated with sLSS; DM also tended to be associated with sLSS. To our knowledge, this is the first report showing a significant association between ABIs and sLSS in the general population.

### Interpretation

Degenerative LSS is defined as a reduction in the space available for the neural and vascular elements of the lumbar spine, secondary to degenerative spinal canal changes [17]. Characteristic sLSS features neurogenic claudication induced during walking or prolonged standing. Thus, mechanisms known to affect posture, including spinal nerve tissue damage or associated blood circulation disorders are thought to be associated with sLSS, although details remain unclear.

Undoubtedly, the anatomically reduced space of the nerve is the most significant factor associated with sLSS development. Therefore, MRI is considered the most appropriate and noninvasive test for confirming narrowed spinal canals and nerve root impingement [17]. Several studies have reported that a dural sac cross-sectional area of <100 mm^2^ indicates central stenosis [19–21]; however, a consensus does not exist for the quantitative radiologic criteria for diagnosing LSS.

Our multiple logistic regression analyses included sLSS confounding factors such as sex, age, BMI ≥ 25, and smoking habits. Although the results were based on semi-quantitative determinations, they indicated that the likelihood of symptomatic disease increased with increasing spinal stenosis severity. This observation corresponds with earlier reports that reduced nerve space is a significant factor predicting sLSS.

An important new finding from this study is that a low ABI is also associated with sLSS. The ABI is used as a diagnostic index for arteriosclerosis obliterans, with the criteria established by the American Heart Association. Normal ABIs range from 0.9 to 1.3, and an ABI < 0.9 has been reported to be diagnostic of PAD [22]. The average ABI associated with intermittent vascular claudication is 0.6 [23]. The mean ABI for individuals in our study was 1.10 (SD, 0.09); when the ABI was 1 SD below the mean, the odds ratio for an individual demonstrating sLSS was 1.24.

Clinically, discriminating between neurogenic claudication associated with LSS and intermittent vascular claudication associated with PAD is critical. Thus, proper diagnosis of buttocks and lower limb symptoms is essential to ensure that intermittent, PAD-associated vascular claudication is correctly diagnosed as LSS. In accordance with the gold standard at the time of this study, orthopedic surgery specialists asked the participants about their symptoms and conducted physical examinations, but the clinical diagnosis of LSS also relied on lumbar MRIs. In our study, 23 participants had ABIs ≤ 0.9, including 6 with ABIs ≤ 0.8, associated with PAD; only 1 participant was diagnosed with LSS. This suggests that 91 of 92 participants did not have PAD in the LSS group. Thus, we believe that the diagnostic accuracy of LSS in this study was high enough and that a low ABI is associated with sLSS.

As suggested above, decreased cauda equina blood flow, because of an anatomic reduction in the spinal canal space available for spinal nerves and vessels, is associated with sLSS. Arteriosclerosis obliterans may impact sLSS by decreasing the cauda equina blood flow, since we know that prostaglandin E1 improves flood flow through peripheral vessels and alleviates sLSS [24,25].

The prevalence of lifestyle diseases, such as hypertension, DM, and PAD, has been reported to be high among individuals with LSS [6, 8, 26, 27]. The prevalence of DM is particularly high (13.6–29.1%) and has been considered a risk factor for LSS. However, the LSS and non-LSS groups in previous studies were compared using bivariate analyses, without factoring age, BMI, or comorbidities. We believe that our results are more reliable because our study design targeted the general population and the analyses were performed taking into consideration several confounding factors that are possibly associated with sLSS. Specifically, the prevalence of DM in the LSS group was 17.4% versus 7.3% in the non-LSS group. When we accounted for sex, age, BMI ≥25, smoking habits, alcohol consumption, ABI, and radiographic LSS grade, only DM showed a significant trend (p < 0.1) toward being associated with sLSS. The results of the stratified analyses in the severe stenosis group failed to show an association between DM and LSS. However, DM was significantly associated with sLSS in the moderate stenosis group. DM and PAD are lifestyle-related diseases and modifiable factors; therefore, we believe that our result will be helpful for the prevention and treatment of LSS.

### Generalization

Although 968 inhabitants were included in this study, they were recruited from only two regions of Japan. Thus, these results may not apply to people living elsewhere in the country or to western populations. However, there was no significant distinction in BMI between the participants in this study and those measured nationally (men, 23.7 [SD, 3.4] vs 23.95 [2.64], respectively; women, 23.1 [3.7] vs 23.50 [3.69], respectively) [28]. On the basis of this comparison, it is feasible that the data demonstrating that low ABIs are associated with sLSS in the inhabitants of the two regions studied applies to the national population.

### Limitations

We acknowledge that this study has several limitations. First, because of the cross-sectional design, we cannot conclude that there is a causal relationship between a low ABI and sLSS. However, this point will be further investigated and clarified in follow-up studies involving the same population. Second, we evaluated LSS using static images. Clinically, dural tube compression, influenced by body position or local instability, is known to be associated with sLSS. Therefore, MRI evaluation or using X-P images in anteflexion while the patient is standing is ideal. However, because the time and resources available are limited in large-scale cohort studies, this will have to be evaluated in a future study.

## Conclusion

The criteria for LSS have not yet been defined, and many of the critical causes remain unknown, except for organic dural tube stenosis/compression. In this study, we demonstrated that rLSS grade and ABI are significantly associated with sLSS. Low ABI is a risk factor for sLSS independent of rLSS. Thus, it is necessary to evaluate ABI in symptomatic LSS patients not responding to treatment. Altogether, these data contribute to the development of improved diagnoses and treatments for patients with LSS.
